# Outcomes of lipectomy in patients with advanced unilateral upper extremity lymphedema with regard to the difference in time required for indocyanine green to reach the axilla: A retrospective cohort study in a single center

**DOI:** 10.1097/MD.0000000000030742

**Published:** 2022-09-16

**Authors:** Ryuck Seong Kim, Changryul Claud Yi, Jae Woo Lee, Jin A Yoon, Seungbeom Lee, Joo Hyoung Kim

**Affiliations:** a Department of Plastic and Reconstructive Surgery, Pusan National University, School of Medicine, Busan, Korea; b Department of Plastic and Reconstructive Surgery, Pusan National University, School of Medicine, Yangsan, Republic of Korea; c Biomedical Research Institute, Pusan National University Hospital, Busan, Korea; d Department of Rehabilitation Medicine, Pusan National University, School of Medicine, Busan, Republic of Korea; e Department of Artificial Intelligence, Pohang University of Science and Technology, Pohang, Gyeongsangbuk-do, Korea.

**Keywords:** lipectomy, lymphatic flow, lymphedema

## Abstract

This study aimed to compare and analyze the prognosis after lipectomy with respect to the difference in time required for indocyanine green (ICG) to reach the axilla in patients with advanced unilateral upper extremity lymphedema. The study population was divided into 2 groups, according to the time required by ICG to reach the axilla after injection, that is, <1 hour (<1 hour; n = 9) and over 1 hour (>1 hour; n = 8). The patient’s arm volume was examined before surgery and up to 12 months after surgery. The volume difference between the 2 groups was compared using the excess volume ratio. Statistically significant differences were not observed before surgery (*P* = .847) and 1 month (*P* = .336), 3 months (*P* = .630), and 6 months after surgery (*P* = .124) between the excess volume ratio values of the < 1 hour and > 1 hour groups. A statistically significant difference was confirmed 12 months after surgery (*P* = .034). The difference in the time when ICG reached the axilla in patients with lymphedema was associated with prognosis after lipectomy. The difference in time could possibly be used as a variable to classify the progress of lymphedema in the future.

## 1. Introduction

Lymphedema is a disease characterized by lymphatic stasis arising from lymphatic damage or dysfunction, which eventually weakens the patient through fat accumulation, fibrosis, and fluid collection.^[1,2]^ Failure to treat lymphedema at an early stage results in a gradual decline in the patient’s quality of life.^[3–6]^ Moreover, the number of patients with lymphedema has increased gradually, owing to increase in the survival rate after cancer surgery with advancements in medical science.^[7]^ However, due to the lack of both awareness of the disease and experts in this field, several patients do not receive adequate treatment and visit the hospital when the disease has reached an advanced stage.^[8,9]^

The goal of surgical treatment for lymphedema is to reduce the accumulated fluid or fat or induce lymphatic drainage through an alternative pathway.^[1]^ Lymphaticovenous anastomosis (LVA) or vascularized lymph node transfer (VLNT) aims to induce physiological lymphatic drainage. In contrast, lipectomy is a surgical method that can be used in patients with advanced fibroadipose hypertrophy and induces reduction.^[10–12]^

Lipectomy can be performed in patients with advanced lymphedema; however, its disadvantage is that controlled compression therapy (CCT) must be maintained for life after surgery.^[11]^ The long-term efficacy of CCT after lipectomy has been confirmed by several studies.^[11,12]^ However, the volume rebound varies for each patient, even if lipectomy and CCT are performed for those with chronic lymphedema. Therefore, we decided to investigate the individual patient-related factors affecting the degree of persistence after surgery, and the time required for indocyanine green (ICG) to reach the axilla was set as a variable in the current study.

Two types of forces are responsible for the flow of lymphatic fluid^[2]^: extrinsic and intrinsic. Extrinsic force is induced by muscle movement, such as the heartbeat, whereas lymphangions play a critical role in intrinsic force. The smooth muscle cells in the lymphatic vessels contract and pump the lymph, which flows along the lymphatic vasculature. A bileaflet valve in the lymphatic endothelial cells prevents reflux to create unidirectional flow.^[2,13]^ Only the intrinsic force was assessed in our study, whereas extrinsic force was excluded from the analysis. The study population was divided into 2 groups based on the time required for ICG to reach the axilla, and each patient’s upper extremity was assessed for the amount of volume reduction maintained after lipectomy.

No study has stratified patients with lymphedema who underwent lipectomy according to time required for ICG to reach the axilla variables and compared their prognoses and outcomes. In this study, the difference in the time of ascent of lymph to the axilla, which was visualized using ICG lymphography, was used as a variable to compare the persistent degree of volume reduction after lipectomy, and an index was suggested to predict the postoperative outcome.

## 2. Methods

This study was conducted after receiving approval from the Institutional Review Board of Pusan National University. The study design was a retrospective chart review, and the study was a retrospective cohort study. This study enrolled patients who were diagnosed with lymphedema of the upper extremity and underwent follow-up for > 12 months after lipectomy between August 2018 and November 2020 in Pusan National University Hospital. The patients’ age, body mass index, and volume of fat removed were confirmed. Patients who developed infection or experienced trauma within 12 months of lipectomy were excluded. Complete decongestive therapy was performed on the patients by the Department of Rehabilitation Medicine for 6 months. During this period, the patients were informed about the practice of CCT. Patients whose symptoms were not improved after the therapy were referred to the Plastic and Reconstructive Surgery Department, and their surgical methods were determined based on their ICG lymphography results.

Lipectomy was performed for patients with modified MD Anderson Cancer Center (MDACC) stage 4 or higher, Arm Dermal Backflow (ADB) stage 4 or higher, and universal ICG stage 3 or higher.^[14–16]^ The circumferences of both extremities were measured before surgery and 1, 3, 6, and 12 months after surgery. ICG dye was injected into the first and third webspaces at the time, the patient visited the outpatient clinic before surgery. Inspections were conducted on both arms, with and without lymphedema. Arm movements were restricted, so that only the intrinsic pathway of lymph flow would come into effect. The patients were instructed to stand in a stationary position with arms at the sides. Most of the patients followed the instructions correctly. Thereafter, images were acquired at intervals of 1 hour, 2 hours, and 3 hours using an infrared camera (Moment K; Gils, Seoul, Korea). Patients in whom ICG did not reach the axilla by 3 hours were evaluated as having advanced stage lymphedema. These data were used to stratify patients into the over 1 hour (>1 hour) and <1 hour (˂1 hour) groups, based on the rate of upward flow of ICG to the axilla.

During the outpatient visit, the compression bandage or garment was removed, and the circumference of the upper arm was measured 10 cm above and below the elbow crease on the right and left sides. Based on the measured circumference, the volume was calculated using the following formula.^[10,17]^


$$\begin{array}{l} V=\pi \times 20\times \frac{R^{2}\times r^{2}\times Rr}{3}, \end{array}$$

$$\begin{array}{l} \;where\;R\;is\;the\;upper\;circumference,\;\;r\;is\;the\;lower\;circumferance,\;\;and\;\pi =3.14 \end{array}$$

The volumes of the affected and nonaffected limbs were calculated before and 1, 3, 6, and 12 months after surgery, respectively.

In order to reduce the bias arising from the change in the patient’s weight or difference in volume according to the measurement time, the difference in the volumes of both limbs was calculated and divided by the volume of the nonaffected limb, which was defined as the excess volume ratio (EVR).^[10]^


$$\begin{array}{l} \text{EVR}=\;\;\;\frac{Va-Vn}{Vn},\;\;\; \end{array}$$

The EVR was calculated at each time point using the values of the limb volumes measured on both sides at 1, 3, 6, and 12 months after surgery.

The EVR at baseline (i.e., preoperatively) was subtracted from the EVR at a given time point to calculate the difference in the EVR over time (relative to the preoperative EVR), which was denoted as the change in the EVR.


$$\begin{array}{l} \left(\text{change }\;\text{ in }\;\text{ excess }\;\text{ volume }\;\text{ ratio}\right)=EVR_{nm}-EVR_{0m},\; \end{array}$$

$$\begin{array}{l} where\;\;\;EVR_{0m}\;is\;the\;\text{EVR }\;\text{ measured }\;\text{ before }\;\text{ surgery }\;\text{ and }\;\;EVR_{nm}\;is\;the\;\text{EVR }\;\text{ measured }\;\text{ n }\;\text{ months }\;\text{ after }\;\text{ surgery} \end{array}$$

Thus, the change in the EVR at 1, 3, 6, and 12 months postoperatively was obtained using these calculations.

The surgical procedure was performed under general anesthesia. An incision measuring approximately 3 mm was placed at the upper limb and tumescent solution was administered using an injection cannula. The suction cannula was connected to the vacuum pump to supply a negative pressure of about 0.9 times the atmospheric pressure. A thin cannula was used to aspirate the distal part, and a relatively thick cannula was used for the proximal part. Liposuction was continued until the thickness of the affected limb was similar to that of the opposite unaffected limb. A compression dressing was applied using an elastic bandage after surgery.^[11,12,18]^

The patients were instructed to retain the compression bandage after surgery and were discharged approximately 3 days after surgery. Patients were asked to use a compression bandage for 1 month after discharge. Subsequently, they were trained to use Mobiderm Autofit (Haddenham Healthcare). Outpatient visits were conducted 2 weeks, 4 weeks, 3 months, 6 months, and 12 months after surgery. To reduce errors in measuring the circumference, patients were requested to visit the outpatient department after placement of the bandage on the day of the visit, and the bandage was removed immediately before circumference measurement.

Volume (cm^3^) was expressed as median and quartiles. The study population was stratified into 2 groups, according to the time required by ICG to rise to the axilla, that is, the ˂1 hour and > 1 hour groups. The volumes of the affected and nonaffected limbs were calculated at each time point. The Wilcoxon rank-sum test was performed to compare the volume of the affected limb of the 2 groups. The EVR was calculated for each time point using the affected and nonaffected limb volumes obtained at that time point. Both groups were subjected to the Wilcoxon rank-sum test. Paired data were compared within each group with the change in the EVR, which was analyzed using the Wilcoxon signed-rank test. *P* < .05 was considered statistically significant. All analyses were conducted using SPSS version 21 for Windows (IBM Corporation, Armonk, NY, USA).

## 3. Results

Nineteen patients underwent lipectomy for lymphedema of the upper extremity and follow-up for 12 months after surgery. A patient with cellulitis and another patient with bilateral lymphedema were excluded. Finally, 17 patients were selected for the analysis. All patients had an advanced stage of disease and had not previously undergone LVA. In addition, there were no patients who underwent VLNT. It was confirmed that ICG reached the axilla within an hour for all arms that had not been affected by lymphedema. Each of the 17 patients developed lymphedema after undergoing surgery for breast cancer. All patients had a history of irradiation. The time required by the ICG dye to reach the axilla was <1 hour in 9 patients and >1 hour in 8 patients. The time taken to reach the axilla was 3 hours in 2 patients in the latter group. There were no statistically significant differences in age, body mass index, and volume of fat removed between the 2 groups. The patients’ characteristics are enumerated in Table 1.

**Table 1:** patient characteristics.

Characteristic	Below 1 hour	Above 1 hour	* *P*
No.	9	8	
Age, yr (median [IQR])	62.00 (55.00–66.00)	59.50 (56.00–65.50)	.9615
Body mass index (median [IQR])	23.8 (23.1–24.6)	24.35 (23.43–25.00)	.8473
Volume of fat removed (median [IQR])	500 (300–600)	550 (437.5–762.5)	.3589

Wilcoxon rank-sum test (

* *P* < .05).

IQR = interquartile range.

The median preoperative volume was 53,985.65 cm^3^ in the group that required >1 hour for ICG to reach the axilla, and 45,804.42 cm^3^ in the group that required < 1 hour; the difference between them was not significant (*P* = .211). Significant differences were not observed at 1 month (*P* = .501), 3 months (*P* = .772), 6 months (*P* = .135), and 12 months (*P* = .083) (Table 2) between the 2 groups.

**Table 2:** Volume of the affected limb stratified by the time to reach the axilla.

	Below 1 hour (N = 9)	Above 1 hour (N = 8)	* *P*
	Median (IQR)	Median (IQR)	
V_affected 0m_	49,448.67 (44,128.90–52,040.48)	53,985.65 (45,479.79–61,727.71)	.211
V_affected 1m_	42,558.11 (37,798.60–49,886.19)	48,126.58 (38,368.01–53,554.34)	.501
V_affected 3m_	40,987.31 (38,594.47–51,082.30)	49,345.26 (39,546.11–51,993.36)	.772
V_affected 6m_	40,903.54 (37,013.20–49,343.95)	52,959.40 (40,563.20–57,535.65)	.135
V_affected 12m_	41,694.17 (38,594.47–48,490.48)	50,747.19 (43,793.80–59,561.98)	.083

Wilcoxon rank-sum test (

* *P* < .05)

IQR = interquartile range, m = month, V = volume.

**Figure 1.**
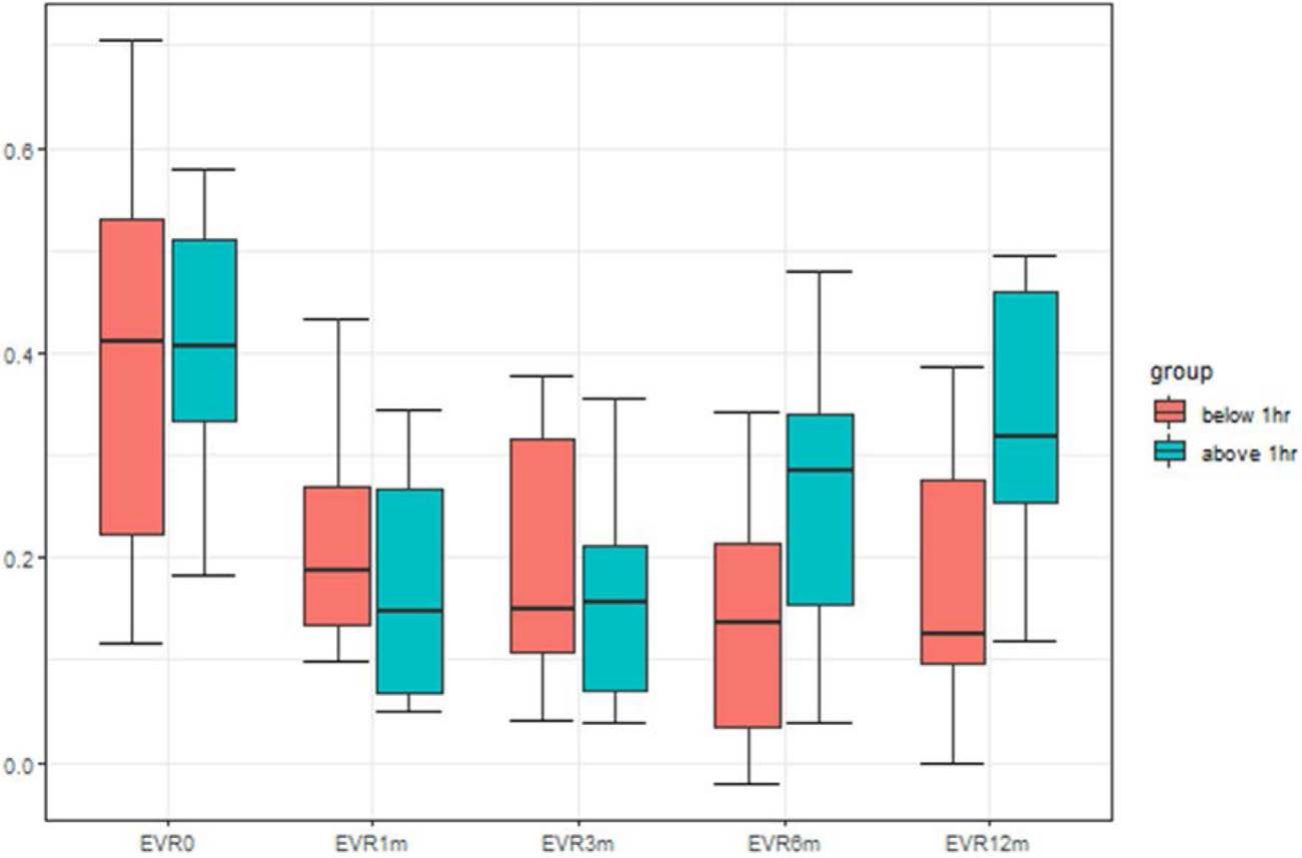
Box plot of EVR values of 2 groups according to time change.

The median of the EVR values in the >1 hour group were as follows: before surgery, 0.41; 1 month, 0.15; 3 months, 0.16; 6 months, 0.28; and 12 months, 0.32. The EVR values in the <1 hour group were as follows: before surgery, 0.41; 1 month, 0.19; 3 months, 0.15; 6 months, 0.14; and 12 months, 0.13. Significant differences between the 2 groups were confirmed at 12 months (Table 3). This trend can be confirmed through the graph (Fig. 1). There was no significant difference between the >1 hour group and ˂1 hour group before surgery. No significant difference was observed between the 2 groups up to 3 months after surgery. However, a difference was observed at 6 months, which attained statistical significance at 12 months (Table 3).

**Table 3:** Excess volume ratio value stratified by time.

	Below 1 hour (N = 9)	Above 1 hour (N = 8)	* *P*
	Median (IQR)	Median (IQR)	
EVR_0m_	0.41 (0.22–0.53)	0.41 (0.33–0.51)	.847
EVR_1m_	0.19 (0.13–0.27)	0.15 (0.07–0.27)	.336
EVR_3m_	0.15 (0.11–0.32)	0.16 (0.07–0.21)	.630
EVR_6m_	0.14 (0.03–0.21)	0.28 (0.15–0.34)	.124
EVR_12m_	0.13 (0.10–0.28)	0.32 (0.25–0.46)	.034

Wilcoxon rank-sum test for continuous variables (

* *P* < .05)

EVR = excess volume ratio, IQR = interquartile range, m = months.

Based on the preoperative EVR value, the difference between the EVR values at 1, 3, 6, and 12 months, respectively, was calculated. The median values were −0.17, −0.22, −0.15, and −0.04 at 1, 3, 6, and 12 months in the > 1 hour group, respectively. The median values were −0.09, −0.12, −0.14, and −0.13 at 1, 3, 6, and 12 months in the ˂1 hour group, respectively. These values were employed to perform intragroup comparison to determine whether there was a significant difference within each group. The results confirmed a significant difference in the EVR values within each group at 1, 3, 6, and 12 months after surgery compared to those before surgery (Table 4).

**Table 4:** Change in the EVR at each time point.

	Below 1 hour		Above 1 hour	
	Median (IQR)	* *P*	Median (IQR)	* *P*
Change_1m_	−0.09 (−0.22 to −0.07)	.004	−0.17 (−0.29 to −0.12)	.008
Change_3m_	−0.12 (−0.16 to −0.08)	.004	−0.22 (−0.30 to −0.14)	.008
Change_6m_	−0.14 (−0.34 to −0.10)	.004	−0.15 (−0.21 to −0.08)	.020
Change_12m_	−0.13 (−0.28 to −0.12)	.008	−0.04 (−0.09 to −0.02)	.008

Wilcoxon signed-rank test for comparison of difference in EVR1m, EVR3m, EVR6m, and EVR12m with baseline (EVR0) (

* *P* < .05).

EVR = excess volume ratio, IQR = interquartile range, m = months.

## 4. Discussion

In this study, patients who underwent lipectomy for lymphedema were stratified into 2 groups according to the time required for ICG to reach the axilla, and the differences between them were compared using statistical tools. No significant difference was detected between the absolute volume of the 2 groups. The lack of a significant difference persisted at 6 months even with volume comparison using EVR. However, the graph in Figure 1 shows that the difference between the 2 groups started to appear at 6 months, which attained statistical significance at 12 months. The results confirmed that the arm volume did not increase relatively well until 12 months in the ˂1 hour group; however, the arm volume in the > 1 hour group was significantly higher than that in the ˂1 hour group at 12 months. Thus, it can be predicted that the prognosis would be relatively poor in the >1 hour group.

The differences in the EVR values were assessed using the change in the EVR at different time points. We found a difference at 12 months in both groups. However, the difference gradually narrowed in the >1-hour group, and we anticipated that there would come a point in time when the change in the EVR would lose statistical significant if observations were to be continued for a prolonged duration.

Although the number of patients was small, there was no significant difference between the 2 groups. First, none of the patients underwent other procedures before lipectomy. Surgical treatment was decided after receiving complete decongestive therapy for 6 months in the department of rehabilitation medicine. After this period, if a patient needed surgery, ICG lymphography would be performed. Thereafter, if it is confirmed to be at an advanced stage, lipectomy would be attempted first. Otherwise, if a patient is at early stage, LVA would be attempted first. Meanwhile, VLNT is attempted when there is no improvement even after lipectomy among patients in the advanced stage. Therefore, none of the patients underwent any other procedures before lipectomy. However, in the case of a patient with breast cancer who received radiation therapy after surgery, the amount of fat removed may be different, possibly resulting in a large bias. However, as shown in Table 1, there was no significant difference in the amounts of fat removed. In addition, there were no differences in the BMI and ages of the patients.

Comparisons were made based on the absolute value of the volume of the arm, but no statistically significant differences were confirmed (Table 2). However, at 6 months a difference began to appear, and the difference had widened further after 12 months. The EVR was used for measurements rather than absolute volume to reduce bias, such as weight changes in patients. Thus, the results of this study are more accurately presented in EVR than in absolute values. Although statistically significant results were not derived from the absolute value of the volume, it was nevertheless meaningful in that it reflected the changes observed in EVR.

Fat accumulation compresses the surrounding lymphatic capillaries, which instigates a vicious cycle, impeding fluid and lipid transport.^[13]^ The > 1 hour group represents a relatively advanced stage of the disease, characterized by a decrease in the lymph flow and delay in the transport of fat and fluid. This aspect is further reflected in the postoperative results; Figure 1 shows the occurrence of rebound at 6 months in the > 1 hour group. Additionally, the analyses in this study confirmed a statistically significant difference in the EVR values of the 2 groups at 12 months (Table 2).

The MDACC, ADB, universal ICG, and International Society of Lymphology staging systems are commonly used to classify patients with lymphedema. Physical examination is used for International Society of Lymphology staging, whereas the MDACC, ADB, and universal ICG staging systems rely on the assessment of back flow after ICG injection.^[15,19]^ In addition to these methods, numerous attempts have been made to determine the severity and prognosis of lymphedema.^[20,21]^ This study statistically confirmed that the arm volume rebounded relatively quickly in the group with slower time required for ICG to reach the axilla in advanced stage lymphedema patients. Our results confirm that the time required for ICG to reach the axilla can be used as an index to predict the outcome of lipectomy. Considering that all patients are patients in the advanced stage, it can be expected that it can act as an index that can complement the abovementioned stage system. However, the previously introduced stage system, such as dermal backflow, cannot divide advanced stage patients in more detail and has limitations in that the time required for ICG to reach the axilla cannot be considered. Therefore, as time required for ICG to reach the axilla was confirmed as a variable that can sufficiently predict the prognosis, it was possible to confirm the possibility that it can be used as an index to predict the outcome after surgery, complementing the stage system.

Technological advancements may enable the accurate measurement of lymph flow velocity, which may help determine the degree of progression and find the exact location of the lymph flow obstruction.^[22]^ If an accurate velocity value is used as an index, it will be possible to more accurately evaluate the prognosis and disease progression.^[22]^ However, since it is impossible with the current technology, an indirect method can be applied as an alternative as in this study. We endeavor to examine the prognostic factors and disease progression of lymphedema according to these differences in time required for ICG to reach the axilla through follow-up studies.

The first limitation of this study is the small sample size. In the case of patients in the advanced stage, compliance was often poor, and several patients could not be followed up until 12 months after surgery. Thus, statistical power was a limitation because the number of patients observed was small. The second limitation is the follow-up period. At 12 months, a statistical difference in EVR could be confirmed; however, no difference could be confirmed in the absolute value of volume. Moreover, change of EVR could not be confirmed after 12 months. However, as a result of the study, it was possible to predict the result after 12 months, but we could not actually confirm it. A more reliable assertion could have been made if the follow-up observation was > 12 months. We intend to verify significant values affecting disease progression through follow-up studies with a larger sample size and longer observation period. The final limitation pertains to the reliability of the volume measurement as some patients may not agree to undergo CCT. Discrepancy may occur depending on whether the patients are compliant with their treatments. However, the patients received complete training regarding the practice of CCT 6 months prior to the surgery in the department of rehabilitation medicine. In addition, if CCT was not performed at the time of visit, the evaluation could not be accurately made. Therefore, all patients were subjected to CCT, when they visited the outpatient clinic, to minimize bias.

Despite the limitations arising from the small sample size and short observation period, this study found that rebound commenced at 6 months, and significant differences were detected in the EVR between the 2 groups at 12 months. There was a significant difference in the increase in volume at 12 months between the group that required >1 hour for ICG to reach the axilla and the group requiring ˂1 hour. These findings can enable the surgeon to provide more information to patients by notifying them of the prognosis before the surgical procedure.

## 5. Conclusion

If it takes a long time for ICG to rise, it can be concluded that rebound occurs relatively faster after lipectomy. On the other hand, if ICG reaches the axilla quickly, prognosis is likely to be relatively good. Thus, patients with a long ICG rise time may be identified as being in the advanced stage.

Our findings suggested that the difference in time may be used as a variable to indicate the progress of lymphedema. Furthermore, follow-up studies are neccessary to create specific criteria that can be used as a stage system. Through this, it is expected that the progression of lymphedema can be accurately monitored based on the time required for ICG to reach the axilla in combination with dermal backflow and physical examination.

## Author contributions

Joo Hyoung Kim—Conceptualization; Ryuck Seong Kim—Writing—original draft; Changryul Claud Yi—Writing—review and editing; Jin A Yoon—Methodology; Jae Woo Lee—Writing—review and editing; Seungbeom Lee—Data curation.
